# In vitro drug release characteristic and cytotoxic activity of silibinin-loaded single walled carbon nanotubes functionalized with biocompatible polymers

**DOI:** 10.1186/s13065-016-0228-2

**Published:** 2016-12-12

**Authors:** Julia Meihua Tan, Govindarajan Karthivashan, Shafinaz Abd Gani, Sharida Fakurazi, Mohd Zobir Hussein

**Affiliations:** 1 Materials Synthesis and Characterization Laboratory, Institute of Advanced Technology (ITMA), Universiti Putra Malaysia, 43400 Serdang, Selangor Malaysia; 2 Laboratory of Vaccine and Immunotherapeutics, Institute of Bioscience (IBS), Universiti Putra Malaysia, 43400 Serdang, Selangor Malaysia; 3 Department of Human Anatomy, Faculty of Medicine and Health Sciences, Universiti Putra Malaysia, 43400 Serdang, Selangor Malaysia

**Keywords:** Anticancer drug, Polysorbate 20, Polysorbate 80, Polyethylene glycol, Chitosan, Surface coatings

## Abstract

In this paper, we demonstrate the preparation of silibinin-loaded carbon nanotubes (SWSB) with surface coating agents via non-covalent approach as an effective drug delivery system. The resulting surface-coated SWSB nanocomposites are extensively characterized by Fourier transform infrared (FTIR) and Raman spectroscopies, ultraviolet–visible (UV–Vis) spectrometry and field emission scanning electron microscopy (FESEM). The FTIR and Raman studies show that an additional layer is formed by these coating agents in the prepared nanocomposites during the coating treatment and these results are confirmed by FESEM. Drug loading and release profiles of the coated SWSB nanocomposites in phosphate buffered saline solution at pH 7.4 is evaluated by UV–Vis spectrometry. The in vitro results indicate that the surface-modified nanocomposites, with SB loading of 45 wt%, altered the initial burst and thus, resulted in a more prolonged and sustained release of SB. In addition, these nanocomposites exhibit a pseudo-second-order release kinetic which was driven by the ion exchange between the ionized SWSB and the anions in the release medium. The cytotoxicity effect of the resulting nanocomposites on normal mouse fibroblast cells is evaluated by 3-(4,5-dimethylthiazol-2-yl)-2,5-diphenyltetrazolium bromide (MTT) assay. It is observed that the surfactant and polymer coating improved the biocompatibility of the SWSB nanocomposites significantly, which deem further exploitation for their application as potential anticancer drug delivery system.

## Background

Cancer, a common name given to a group of related illnesses, has a great impact on public health across the world. In the United States, cancer is the second leading cause of death after heart disease, accounting for nearly 1 of every 4 deaths [1]. According to the source which was published recently, American men have a slightly higher risk for developing cancer (less than 1 in 2) compared to women (a little more than 1 in 3) over the course of their lifetimes. These figures reveal that, cancer rates are growing at an alarming speed and it is expected to rise by 57% globally in the next 20 years, as predicted by World Health Organization [2].

Chemotherapy is the drug treatment for cancer disease using powerful chemicals, and it is expected to kill the cancer cells for maximum treatment efficacy without destroying other normal cells in the body. However, many of the conventional chemotherapies are often associated with drug administration problems like lack of selectivity, limited solubility, poor distribution, systemic toxicity and the inability of drugs to cross cellular barriers. Therefore, it is essentially important for medicinal chemists to alter the drug actions by developing a well-designed drug delivery system with specific tumour-targeting and pH-triggered unloading properties, while reducing unwanted side effects (e.g. fatigue, nerve damage, nausea, hair loss, skin and nail changes, heart trouble, and etc.) which can lead to serious complications.

In the recent years, silibinin (SB) has received a great amount of attention as herbal remedy to treat cancer-related diseases. It has demonstrated potential clinical applications in the treatment of neurodegenerative and neurotoxic related diseases, diabetes mellitus, Amanita mushroom poisoning, several types of nephrotoxicity, alcoholic liver cirrhosis and various forms of in vitro and in vivo cancer models [3–6]. SB, as the main constituent of silymarin, is obtained from the medicinal plant *silybum marianum* (milk thistle) and has been used for centuries to treat liver disorders due to its potent hepatoprotective effect [7]. However, its low solubility in aqueous environment which leads to poor bioavailability in the human body, has limited its clinical potential in biomedical applications.

Carbon nanomaterials such as carbon nanotubes have been extensively researched as a carrier for anticancer drugs [8], as they are capable of penetrating cellular membranes [9] and allow for high drug loading [10] due to their unique architectural features (e.g. high aspect ratio and nanoscale dimensions). They have the potential to deliver therapeutic molecules to the targeted site of action by conjugation to ligands of cancer cell surface receptors or antigens [11], which makes them an ideal delivery system to treat cancer diseases at the cellular level. In addition, they can be covalently or non-covalently functionalized with hydrophilic materials such as polysorbate surfactant and polyethylene glycol (PEG) [12, 13], to improve their biocompatibility and dispersability in physiological environment.

Previously, we have reported the preparation of SB-loaded nanohybrid based on carboxylic acid functionalized single walled carbon nanotubes (SWCNT-COOH) [14]. Our preliminary findings showed that the system, with low toxicity, significantly suppressed the growth of human cancer cell lines, in particular, human lung cancer cells (A549) when compared to pure SB. Furthermore, the system possess favourable sustained release characteristic and the release rate is pH-dependent which further justify its potential to be developed into novel drug delivery system for cancer treatment. In this work, as an attempt to further improve the system’s biocompatibility, we have designed and prepared a new type of drug delivery system involved the use of surface-modified SWCNT for water-insoluble anticancer drug, SB. Biocompatible surface coating agents, namely polysorbate 20 (T20), polysorbate 80 (T80), PEG and chitosan (CS) were used to non-covalently wrapped around the SB-loaded SWNTs (SWSB), imparting water-solubility and biocompatibility to the nanotubes.

Normal mouse fibroblast cells (3T3) were employed to be comparable to the existing peer-reviewed literature since a vast number of papers suggest that carbon nanotubes possess a potential toxicological effect [15–17] but little is known about the cytotoxicity of drug-loaded carbon nanotubes, particularly of SWCNT form. In general, fibroblasts are the most versatile of connective-tissue cells and form supporting framework (stroma) of tissues through their secretion of extracellular matrix components which consists of ground substance and fibres [18]. Besides, these connective tissues play a critical role in wound healing and fibrosis, sharing some similarities with cancer-associated fibroblasts that are present within the tumour stroma of many cancers [19]. For this purpose, the biocompatibility and cytotoxicity characteristic of surface-coated SWSB in fibroblasts were investigated by the 3-(4,5-dimethylthiazol-2-yl)-2,5-diphenyltetrazolium bromide (MTT) assay under in vitro environments.

## Experimental

### Materials

The SWCNT-COOH of purity 90 wt% (impurities: <5% metal oxide as determined by TGA) and produced by the method of chemical vapour deposition, was purchased from Chengdu Organic Chemicals Co., Ltd. (Chengdu, China). They consist of short carboxyl carbon nanotubes with a diameter of 1–2 nm and a length of 1–3 μm (thus, aspect ratio >1000) and the COOH content was found to be around 2.73 wt%. The SB (≥98% purity, 482.44 g mol^−1^) and ethanol (>99.8% purity) were purchased from Sigma-Aldrich (Buchs, Switzerland) and the latter was used as solvent for SB. The T20 (polyethylene glycol sorbitan monolaurate, C_58_H_114_O_26_), T80 (polyethylene glycol sorbitan monooleate, C_64_H_124_O_26_), CS (low molecular weight, 75–85% degree of acetylation) and phosphate buffered saline (PBS) solution were sourced from Sigma-Aldrich (Saint Louis, USA). PEG (average molecular weight 300) was supplied by Acros Organics (Geel, Belgium). Acetic acid (99.8% purity) was obtained from HmbG Chemicals (Hamburg, Germany) and used as solvent for CS. All materials were analytical reagent grade and used without further purification.

### Instruments

Fourier transform infrared (FTIR) measurements were performed on a Thermo Nicolet Nexus 671 (model Smart Orbit). The FTIR spectra of the samples were recorded in the scanning range of 400–4000 cm^−1^ with 32 scans at a resolution of 2 cm^−1^ using KBr disc method, except for pure T20 and T80 via a direct deposition method. Raman spectra were collected using a WITec UHTS 300 Raman spectrometer with an excitation wavelength at 532 nm and detailed scans were performed in the range of 100–3000 cm^−1^. UV–Vis spectra were used to study the optical property of the samples in a drug release experiment, using a Perkin Elmer Lambda 35 spectrophotometer. Thermogravimetric analysis (TGA) was carried out using a TA Q500 with a heating rate of 10 °C min^−1^ under a nitrogen purge of 40 mL min^−1^ in the temperature range of 30–900 °C. The coating content was calculated to be about 19.3, 56.4, 15.7 and 4.6 wt% for T20, T80, PEG and CS respectively, based on the comparison of coated samples with the uncoated ones [20]. The surface morphology of the samples was captured on a Hitachi UHR SU8030 FESEM at 10 kV.

### Preparation of carbon nanotubes-silibinin formulation (SWSB)

The solution of SB was prepared according to the method described by our previous report [14]. It is noted that the best-fit linearity was obtained in the range of 0.003–0.05 mg mL^−1^ in ethanol and thus, maximum dosage of SB at 0.05 mg mL^−1^ was selected in the study. Approximately 400 mg of SWCNT-COOH (as the starting material) was incubated in 400 mL of SB solution and sonicated in a water bath for 1 h in order to separate the nanotubes. Subsequently, the pH of the suspension was slowly adjusted to 4 to facilitate SB uptake. The suspension was then magnetically stirred at room temperature for about 20 h and followed by a centrifugation step at 4000 rpm for 15 min. After discarding the supernatant, the nanotubes were washed three times with ethanol and deionized water in order to remove excessive unbound SB. Finally, the product was dried in an oven at 60 °C for 24 h to obtain SWSB.

### Preparation of the surface-coated SWSB nanocomposites

The surface-coated SWSB was synthesized by adding 100 mg of SWSB into 100 mL of deionized water containing 1% T20, T80, PEG or 0.5% CS (v/v) and magnetically stirred for 24 h at room temperature. After that, the reaction mixture was then collected, centrifuged and rinsed with deionized water three times. Finally, the black precipitate was left to dry completely in an oven to yield SWSB-T20, SWSB-T80, SWSB-PEG or SWSB-CS nanocomposites.

### Drug loading and releasing

The amount of SB loaded into the SWCNT-COOH was determined by measuring the absorbance at 288 nm relative to a calibration curve based on the wt% of the initial drug to the unbound drug in the supernatant using a UV–Vis spectrophotometer. The drug loading capacity of SWCNT-COOH with SB was calculated to be around 45 wt%. Orally administered SB is known to demonstrate low oral bioavailability of 30–50% due to rapid metabolism of the first-pass effect to form conjugates such as glucuronide and sulfate which may not have the same biological activities as the parent compound [21, 22]. Since the loading of SB in the prepared carbon nanotubes was within the bioavailability range of the drug and hence, this concentration (about 45 wt% of loaded SB) was used throughout the study.

To examine the drug release behaviour, 1 mg of surface-coated SWSB was dispersed in 3.5 mL of PBS release media at pH 7.4 (simulating human body physiological condition). The temperature inside the UV–Vis machine was found to be approximately ±35 °C. The release amount of SB was recorded at predetermined time intervals and the release data was then fitted into five kinetic mathematical equations (i.e. zero order, pseudo-first order, pseudo-second order, Higuchi and Korsmeyer-Peppas models).

### Cell culture conditions

Cytotoxicity experiments were performed on the normal mouse fibroblast cell line 3T3 (ATCC, Manassas, USA). The cells were maintained as monolayers in plastic flasks in DMEM supplemented with 10% fetal bovine serum, 15 mmol L^−1^ l-glutamine, 100 units mL^−1^ penicillin, and 100 g mL^−1^ streptomycin and grown in a humidified incubator with 5% CO_2_ at 37 °C. Confluent cells were trypsinized in a trypsin/EDTA solution and subsequently seeded into a 96-well plate containing 1 × 10^5^ cells mL^−1^ and kept overnight for cell attachment. For treatment purpose, old media were discarded and new culture medium (controls) or culture medium containing different concentrations of surface-coated SWSB was added to the wells for 24 h. Suspensions of the coated samples were freshly prepared in PBS medium. Prior to the cytotoxicity experiment, the stock suspension was ultrasonicated in 10 s sequential steps for a total time of 30 s in order to reduce agglomeration. The suspensions were prepared by diluting to the desired concentrations of 3.125, 6.25, 12.5, 25, 50, 75, and 100 μg mL^−1^.

### MTT cytotoxicity assay

The MTT assay, which converts viable cells with active metabolism into a purple coloured formazan, was used to measure cell viability in 3T3 cell line. After culturing overnight, the cells were treated with different concentrations of SWSB-T20, SWSB-T80, SWSB-PEG and SWSB-CS in freshly prepared PBS medium and the plates were incubated at 37 °C in a 5% CO_2_ humidified incubator for 72 h. Following incubation, 20 μL of MTT was added to each well and the plates were incubated for another 3 h. Subsequently, the solution in each well containing excessive MTT and dead cells was discarded, and 100 μL of detergent reagent (dimethyl sulfoxide) was then added to the cells to stop the conversion and solubilize the formazan. The quantity of formazan formed is directly proportional to the number of viable cells after the treatment. The absorbance was measured at 570 nm using a microplate reader (Model EL 800X), with 630 nm as reference wavelength and the obtained data were averaged and fitted to Eq. 1, to determine the percentage of cell viability. The cells cultured without nanotubes were used as control. The experiment was performed in triplicate, and the result was expressed as the percentage of cell viability with respect to control cells.

1 $${\text{Cell viability }}\left( \% \right) = \left( {{\text{OD}}_{\text{treatment}} - {\text{OD}}_{\text{medium}} } \right)/\left( {{\text{OD}}_{\text{control}} - {\text{OD}}_{\text{medium}} } \right) \, \times { 1}00$$

where OD = optical density.

### Statistical analysis

Cytotoxicity data in 3T3 cells were obtained from independent experiments with *n* = 3 for each data point. All data were expressed as the mean and standard deviation (±SD) and compared by one-way analysis of variance (ANOVA) and t-tests using SPSS version 20.0 software.

## Results and discussions

### Fourier transform infrared

**Fig. 1:**
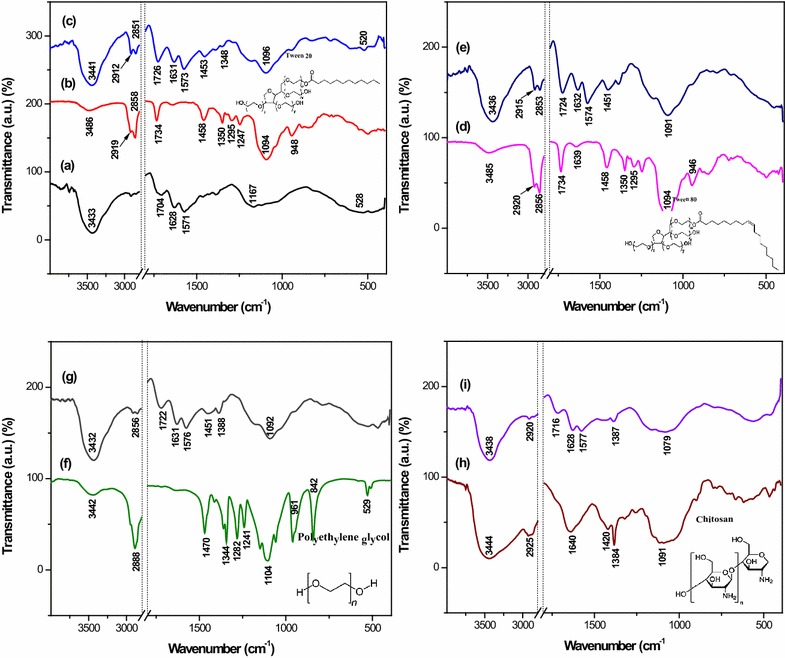
FTIR spectra of (*a*) SWSB, (*b*) T20, (*c*) T20-coated SWSB, (*d*) T80, (*e*) T80-coated SWSB, (*f*) PEG, (*g*) PEG-coated SWSB, (*h*) CS and (*i*) CS-coated SWSB along with their chemical structures

The characteristic bands of SWCNT-COOH, SB and the final product, SWSB (Fig. 1a) have been discussed in our previous paper and therefore, in this work the emphasis is being placed on the surface-coated SWSB nanocomposites. The FTIR spectrum of pure T20 in Fig. 1b demonstrated two strong bands at 2919 and 2858 cm^−1^ that could be due to the asymmetric and symmetric C–H stretching vibrations of the methylene (CH_2_) group [23]. The absorption bands at 1458 and 1350 cm^−1^ are attributed to the asymmetric and symmetric C–H bending vibrations of the methyl (CH_3_) structural unit in the T20 [24]. The other characteristic bands occurred at 3486 and 1734 cm^−1^ are assigned to the O–H vibration of the hydroxyl group or adsorbed water and C=O stretching of the ester group, respectively. All these peaks were seen to be shifted to lower wavenumber in the SWSB-T20 nanocomposite (Fig. 1c), which show that significant interaction has taken place between T20 and SWSB. Since the chemical structure of T80 (Fig. 1d) is similar to that of T20, the relative intensities of those characteristic absorption bands are also observed in the SWSB-T80 nanocomposite (Fig. 1e).

Figure 1f and g are the FTIR spectra of pure PEG and SWSB-PEG, respectively. The FTIR spectrum of PEG (Fig. 1f) demonstrates that the most intense absorption band at 1104 cm^−1^ is due to the functional group of carbon oxygen (C–O) single bond of primary alcohol. The peaks occurred at 3442, 1344 and 529 cm^−1^ are attributed to the O–H stretching vibrations, while the absorptions observed in the region 961 and 842 cm^−1^ correspond to the C–C–O asymmetric stretch and C–C–O symmetric stretch, respectively. Also, the IR peaks at 2888 and 1470 cm^−1^ are due to the C–H stretching and bending vibrations in PEG [25]. For the case of SWSB-PEG (Fig. 1g), some of the bands disappeared, and the others were shifted to the lower frequency due to the chemical interaction between the PEG and SWSB. For example, the peak at 529 cm^−1^ due to the O–H vibration disappeared, and in addition, two new peaks were formed at 1451 and 1388 cm^−1^ which are assigned to the CH_2_ bending and COO^−^ symmetric stretch, respectively.

The FTIR spectrum of pure CS (Fig. 1h) presents a strong band at 3444 cm^−1^ indicative of asymmetric NH_2_ and O–H stretching vibration, while absorption bands at 2925, 1420 and 1384 cm^−1^ are due to typical C-H bond in –CH_2_ and –CH_3_ symmetrical deformation mode, respectively. The sharp band occurred at 1640 cm^−1^ is related to the characteristic of carbonyl bonds (C=O) of the amide group and the band at 1091 cm^−1^ corresponds to the stretching vibrations of C–O from C–O–C bonds [26]. In the spectrum of SWSB-CS (Fig. 1i), most of the bands are belong to CS functional groups and the –OH stretching frequency was seen to be shifted from 3444 to 3438 cm^−1^. This could be due to the ionic π bonds interaction between the CS and the nanotubes, which is consistent with previous report [27].

### Raman

**Fig. 2:**
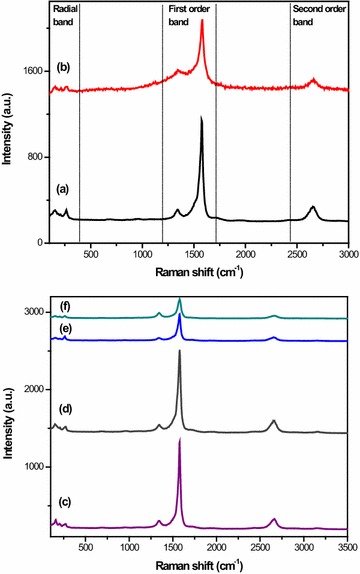
Raman spectra of (*a*) SWCNT-COOH, (*b*) SWSB, (*c*) SWSB-T20, (*d*) SWSB-T80, (*e*) SWSB-PEG and (*f*) SWSB-CS nanocomposites

The Raman spectra of surface-coated SWSB are shown in Fig. 2c–f, while the Raman spectra of SWCNT-COOH and uncoated SWSB have also been included in Fig. 2a, b for the purpose of comparison. There are three distinct bands to be observed in the Raman spectrum of SWCNT-COOH. The presence of the R-band (radial breathing mode) in the low frequency range between 100 and 300 cm^−1^ is dependent upon the tube diameter and this region varies with different samples. In the first order band region, two Raman bands are observed: the band occurred at 1342 cm^−1^ is generally known as the disorder-induced D-band and a higher intensity band centered at 1575 cm^−1^ is often called the tangential G-band. The D-band is correlated with structural defects and disorder present in the graphitic sp^2^ carbon systems, whereas G-band is closely related to the planar vibrational mode of sp^2^-bonded carbon atoms on the graphitic surface of the nanotubes [28]. The second order G’-band near 2650 cm^−1^, which appears in the phonon spectra of sp^2^ carbon-based materials, corresponds to the overtone of the D-band. It is observed that the Raman spectra are very similar for all samples (Fig. 2a–f), suggesting that the nanotubes structure remains unmodified by the coating treatment of the polymers.

The degree of functionalization and imperfections can be estimated by measuring the intensity ratio (I_D_/I_G_) of the D and G-band of the nanotubes [12]. The positions of D and G-bands as well as I_D_/I_G_ ratios for all samples are listed in Table 1. It is found that the I_D_/I_G_ ratio increases after functionalization with SB, and as expected, this value was seen to be decreased gradually after coating treatment. However, this is not the case for CS-coated SWSB. This could be possibly due to the little amount of CS used in the present study which resulted in promoting more defects on the surface of the nanotubes when compared to the others. On the other hand, it is observed that the Tween series have slightly lower defect concentrations, indicating that both T20 and T80 have the best surface wrapping on SWSB. Furthermore, it is worth to be noted that, the intensity ratio of I_D_/I_G_ changes slightly from 0.550 for SWSB to 0.231–0.602 for coated samples, suggesting that the coating process occurred through a non-covalent interaction. This is because a covalent functionalization would have significantly increased the I_D_/I_G_ ratio to >1 [29].

**Table 1:** Peak positions of D and G-bands as well as I_D_/I_G_ ratios for SWCNT-COOH, SWSB and the surface-coated nanocomposites

Sample	D-band (cm^−1^)	G-band (cm^−1^)	Intensity ratio (I_D_/I_G_)
SWCNT-COOH	1342	1575	0.273
SWSB	1338	1575	0.550
SWSB-T20	1346	1579	0.231
SWSB-T80	1346	1579	0.241
SWSB-PEG	1342	1579	0.434
SWSB-CS	1342	1579	0.602

### Field emission scanning electron microscope (FESEM)

**Fig. 3:**
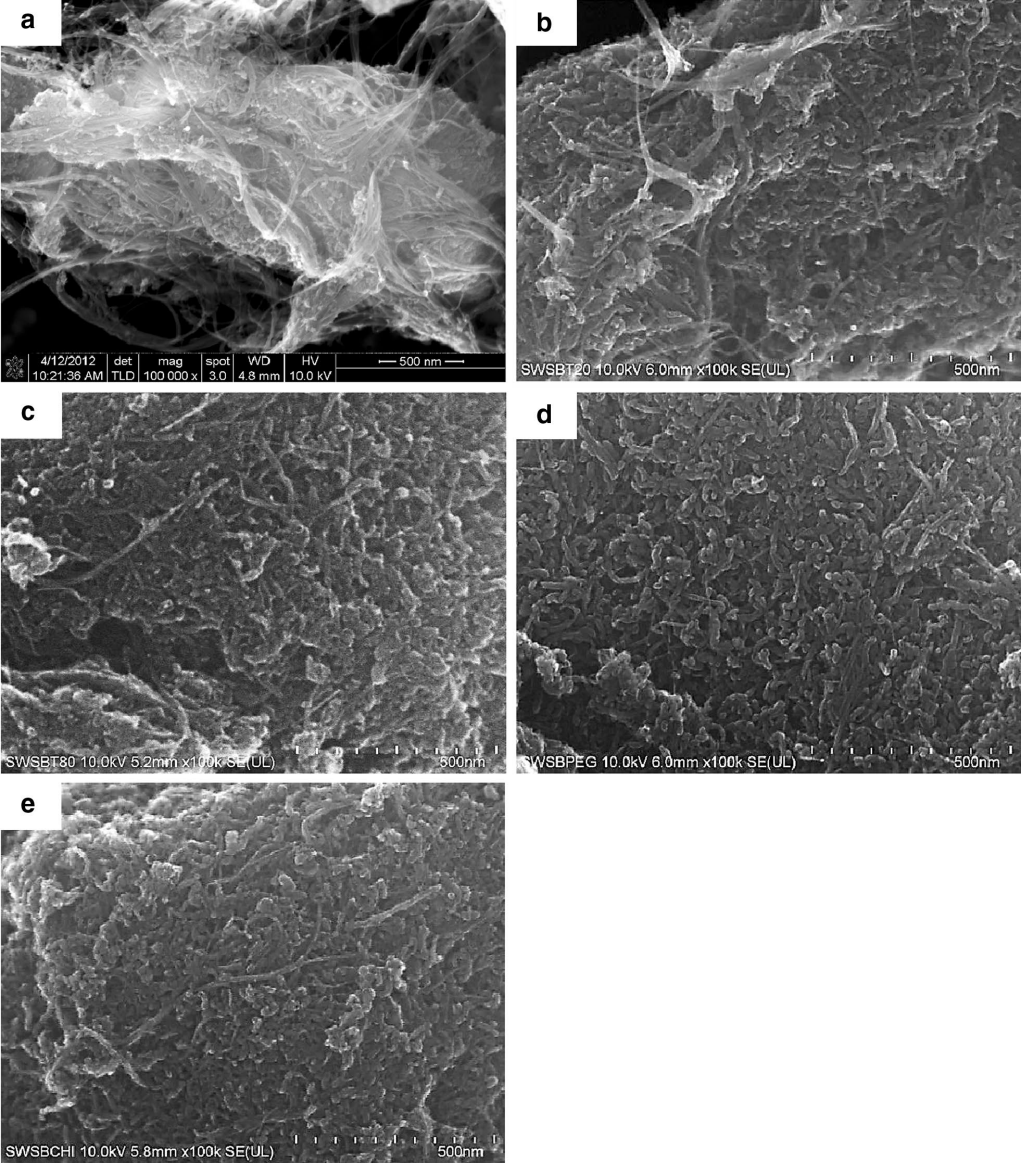
FESEM images of (**a**) SWCNT-COOH, (**b**) SWSB-T20, (**c**) SWSB-T80, (**d**) SWSB-PEG and (**e**) SWSB-CS at magnification 100 k×

FESEM has been used to study the surface morphology of the surface-coated SWSB nanocomposites (Fig. 3b–e), with SWCNT-COOH used as the comparison (Fig. 3a). SWCNT-COOH was seen to be appeared in bundles due to van der Waals interaction with smooth tubular surface structure. After coating of the SWSB with polymers, the surface morphologies of the nanotubes were significantly different from the starting material. Therefore, we inferred that the polymers assist in the dispersion and wrapping of the SWSB by covering most of the surface defects of the nanotubes and hence, a more compact structure of nanocomposites was observed. (e)

### Drug release behaviour at pH 7.4

**Fig. 4:**
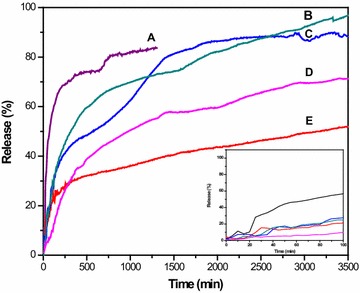
Release profiles of SB from (*A*) SWCNT-COOH, (*B*) SWSB-PEG, (*C*) SWSB-T80, (*D*) SWSB-CS and (*E*) SWSB-T20 at pH 7.4 with maximum release rate of 84, 99, 91, 73 and 58% respectively. *Inset* shows the initial release of the nanocomposites at pH 7.4 in the first 100 min

In our previous work, we have demonstrated that the system (SWSB) released SB in a pH-dependent fashion, with the maximum release of approximately 84% in pH 7.4 as compared to 56% in pH 4.8. However, at the beginning stage of the drug release, we observed a fast release near to 47% after 60 min and then followed by a slower step of sustained release up to 1300 min. As an attempt to reduce the initial burst, we have coated the system separately with different types of polymers and then study the coating effect on the drug release behaviour in PBS solution at pH 7.4. Figure 4 illustrated the release profiles of SB from the surface-coated SWSB nanocomposites, with SB loading of 45%, based on the UV–Vis measurement. After the coating process, the release rate of SB from the coated nanocomposites (Fig. 4b–e) was significantly lower than the release rate of SB from the uncoated ones (Fig. 4a), with the amount of initial release reduced to approximately 6–17% after 60 min. This is because the surface coating molecules formed an additional layer by wrapping around the nanotubes [30], providing extra protection to the encapsulated SB from instant release at pH 7.4 environment and as a result, the release rate of SB was reduced. Due to the presence of the coating agents, the release of SB from coated samples could still be observed even after 3500 min with a slow and sustained release characteristic. As SB is a drug characterized by its relatively short elimination half-life of 4–6 h [31] due to poor absorption in the body, hence, the slow and sustained release behaviour of SB with a release time of more than 48 h may greatly benefit the anticancer treatment.

It is observed that the release behaviour of SB from the surface-coated systems follows a specific order of SWSB-PEG > SWSB-T80 > SWSB-CS > SWSB-T20, as demonstrated in Fig. 4b–e. Among the systems, SWSB-PEG was found to exhibit the highest release rate due to the hydrophilic nature of the PEG molecules which enhances the solubility of hydrophobic carriers (e.g. SWCNT-COOH) and drugs (e.g. SB) in aqueous environment, as a result of the steric hindrance [32]. Interestingly, remarkable differences were also noted in the release behaviour of SB from the nanocomposites coated by Tween surfactants. For example, SWSB-T80 demonstrated a higher release rate of 91% compared to the release rate of 58% from SWSB-T20 at the end of the experiment. This is because partial hydrolysis of ester groups occurred at pH 7.4 which causes the polymeric chains in T20 and T80 underwent ionization, thereby producing more charged –COO^−^ groups. The polymeric systems would then encounter different extent of swelling due to the repulsion forces between the ionized carboxyl groups [33], thus causing the drug molecules to be diffused through water-filled outermost layer at a different rate. As for the SWSB-CS, the released SB from the system was nearly 73%, even though it has the least coating content of 4.6 wt% as measured by TGA analysis. Under the neutral environment (pH 7.4), the hydrophilic carboxyl groups from SWCNT-COOH will be ionized [34], facilitating the release of SB from the surface of nanotubes into the CS thin coating. As a result, the CS polymer swelled causing a constant slow diffusion of SB molecules into the PBS medium. The in vitro drug release experiments showed that the drug release behaviour can be altered by using various selections of biocompatible polymers to suit different therapeutic applications.

### Drug release kinetics and possible mechanisms

To study the release kinetics of SB, data obtained from in vitro drug release experiments (Fig. 4) can be fitted into five different mathematical kinetic models [35, 36] as shown in Table 2.

**Table 2:** Linear regression analysis (R^2^) of samples and their corresponding overall mean percent error (MPE) obtained by fitting the SB release data from biocompatible surface-coated SWSB nanocomposites into PBS solution at pH 7.4

Model name	**Equation**	**Sample**	**R** ^**2**^	**MPE**
Zero-order	$q_{t} = q_{0} + k_{0} t$	SWSB^a^	0.9367	0.0172
		SWSB-T20	0.9914	0.0662
		SWSB-T80	0.6977	0.3247
		SWSB-PEG	0.9631	0.3080
		SWSB-CS	0.9120	0.3926
Pseudo-first-order	$\ln (q_{e} \text{ - }q_{t} ) = \ln q_{e} - k_{1} t$	SWSB^a^	0.9533	8.0461
		SWSB-T20	0.9933	0.3574
		SWSB-T80	0.9402	1.6279
		SWSB-PEG	0.9797	1.6844
		SWSB-CS	0.9720	0.9793
Pseudo-second-order	$\frac{t}{{q_{t} }} = \frac{1}{{k_{2} q_{e}^{2} }} + \frac{t}{{q_{e} }}$	SWSB^a^	0.9983	1.0189
		SWSB-T20	0.9903	1.5389
		SWSB-T80	0.9856	0.3775
		SWSB-PEG	0.9924	1.1613
		SWSB-CS	0.9948	0.3431
Higuchi	$q_{t} = K_{H} \sqrt t$	SWSB^a^	0.9628	0.1231
		SWSB-T20	0.9968	0.1841
		SWSB-T80	0.8966	8.4337
		SWSB-PEG	0.9774	3.0315
		SWSB-CS	0.9583	6.4891
Korsmeyer-Peppas	$\frac{{q_{t} }}{{q_{\infty } }} = Kt^{n}$	SWSB^a^	0.9542	0.0067
		SWSB-T20	0.9793	0.0071
		SWSB-T80	0.9283	0.0022
		SWSB-PEG	0.9612	0.0028
		SWSB-CS	0.9053	0.0391

^a^Release of SB was limited to 1300 min*. q*_*t*_, *q*_*e*_ and *q*_*∞*_ refer to the amount of drug released at time (*t*), at equilibrium and at infinite time. *k*_*0*_, *k*_*1*_, *k*_*2*_ and k_H_ are rate constant of the models

**Fig. 5:**
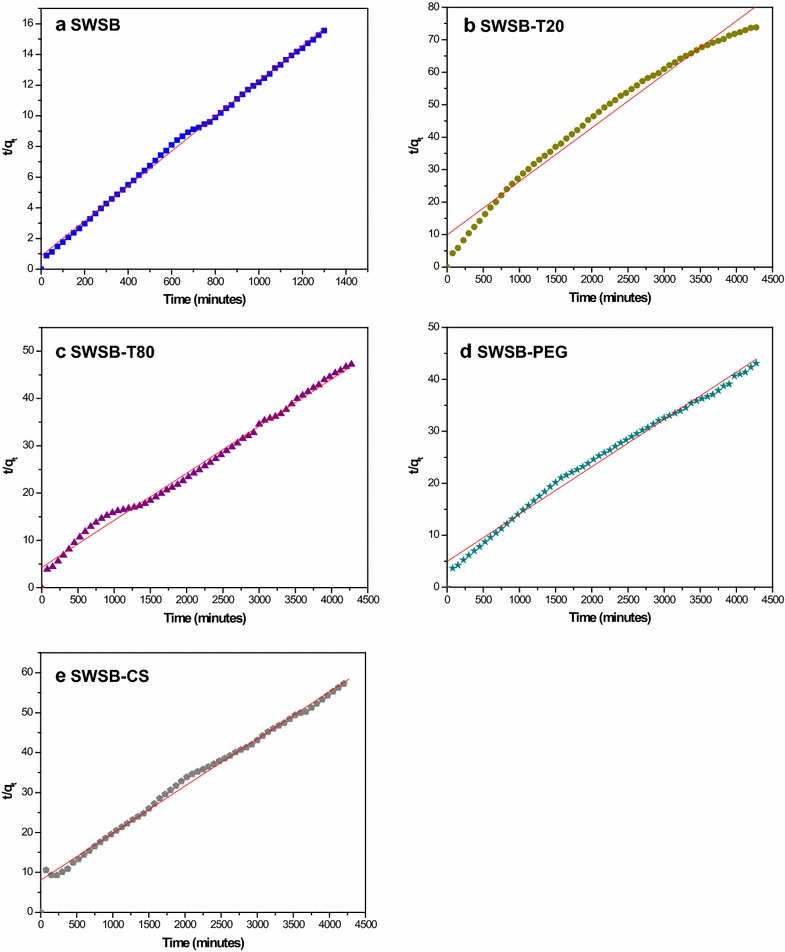
Fits of the release data of SB from (**a**) SWSB, (**b**) SWSB-T20, (**c**) SWSB-T80, (**d**) SWSB-PEG and (**e**) SWSB-CS at pH 7.4 using pseudo-second-order kinetic model

Based on the release kinetics data listed in Table 2, the pseudo-second-order kinetic model with the best linear fit was found to be more appropriate for depicting the release kinetic processes of SB from the surface-coated nanocomposites (Fig. 5). This indicates that the rate limiting step may be chemisorption involving the exchange of electrons between the surface-coated SWSB and the anions in the PBS medium at time of release and that released at equilibrium.

### Effects of surface-coated SWSB on cell viability

**Fig. 6:**
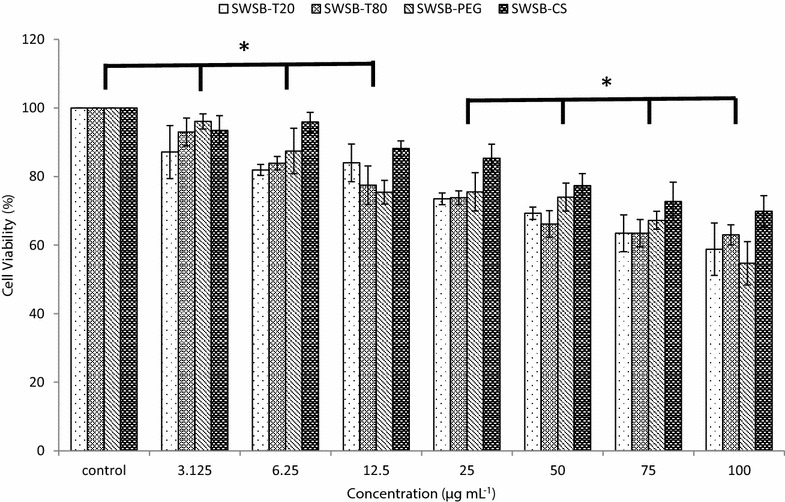
Cell viability of 3T3 cell line treated with SWSB-T20, SWSB-T80, SWSB-PEG, and SWSB-CS for 72 h. Cell viability is calculated as a percentage of absorbance of treated cells over absorbance of untreated cells. Data are shown as mean ± standard deviation from three separate experiments (*n* = 3).* Asterisks* indicate statistically significant differences of the cell viability between the concentrations (p < 0.05)

Most cytotoxicity research in the literature has used the concentration range of carbon nanotubes between 0.1 and 200 μg mL^−1^ with maximum incubation up to 96 h on different types of normal cell lines [37–40]. This is because carbon nanotubes is generally associated with a concentration- and time-dependent increase in cell death as investigated by the use of the MTT assay. Therefore, in the present work, healthy 3T3 fibroblast cell line was used to treat with various doses ranging from 3.125 to 100 μg mL^−1^ of surface-coated SWSB for 72 h and the effect of polymer coatings on cell viability was evaluated by MTT assay (Fig. 6).

Although a vast number of studies have demonstrated that the surfactants and polymers are non-toxic, as they are capable of enhancing dispersibility to promote biocompatibility, still, it is essential to investigate the effect of the conjugation on healthy cells. The cytotoxicity results showed that the coating agents have tremendously improved the biocompatibility of SWSB nanocomposites in comparison with our previous findings [14], in which the non-coated compounds demonstrated cytotoxicity when the concentration exceeded 25 μg mL^−1^. In particular, the uncoated SWSB at concentration of 50 μg mL^−1^ showed 20.6% viability, whereas the coated SWSB showed 69.3, 66.2, 73.9 and 77.3% viability for T20, T80, PEG, and CS, respectively. However, it was seen that the surface-coated SWSB nanocomposites demonstrated a gradual decrease in the cell viability as the concentration increases, with the lowest cell viability of 54.7% observed in SWSB-PEG at concentration of 100 μg mL^−1^. The low viability of PEG-coated SWSB could be attributed to the toxic substances (i.e. monomer, dimer, and trimer), impurities (e.g. fatty acids, catalyst residue, ethylene oxide) and by-product (e.g. 1,4-dioxane) present in the low-molecular-weight glycol used in this study [41–43]. These in vitro results reveal that the surface coating agents expressed different level of cytotoxic effects to the normal mouse fibroblast cells and therefore, further investigation in terms of specific cellular mechanism is deem necessary in order to elucidate the mode of interactions with normal human fibroblasts and cancer-associated fibroblasts within different tumours.

## Conclusions

We demonstrated the preparation of surface-coated SWSB nanocomposites through a simple non-covalent method. In order to achieve better dispersion and improved biocompatibility, T20, T80, PEG and CS were used as a coating agent separately. FTIR and Raman studies confirmed the chemical interaction between the biocompatible polymers and SWSB. The release of SB from the surface-modified system occurs only after water penetration in the polymeric outer layer, followed by diffusion process to the surface of the system. Furthermore, the release of SB is correlated to the swelling characteristics of the surfactants. Despite the structural similarity between T20 and T80, the mechanisms of release are distinctively different, with the higher release rate observed in SWSB-T80 (~91%) compared to SWSB-T20 (~58%). In addition, the released SB from the coated systems is described by pseudo-second-order release mechanism, and that the release fashion is a slow and sustained process which may benefit the anticancer treatment significantly. The in vitro cytotoxicity study shows that the coating agents greatly enhanced the dispersibility and biocompatibility of the SWSB, with an increase of approximately 48.7% (SWSB-T20), 45.6% (SWSB-T80), 53.3% (SWSB-PEG), and 56.7% (SWSB-CS) viability at 50 μg mL^−1^ as compared to the uncoated ones. However with cell viability assays, it would be difficult to draw accurate and reliable conclusions as these nanotubes might potentially interfere with viability markers in the assay systems, leading to a false positive or false negative result of cell viability. As such, several different spectrophotometric assays such as lactate dehydrogenase (LDH) leakage, water soluble tetrazolium salts (WST-1) and [2,3-bis-(2-methoxy-4-nitro-5-sulfophenyl)-2H-tetrazolium-5-carboxanilide] (XTT) should be used in conjunction with MTT assay for this new class of nanomaterials. Nonetheless, this work is a good preparation for our following research on the in vitro cellular mechanism study to assess how they interact with cells.
